# Tenofovir alafenamide versus entecavir for treating hepatitis B virus-related acute-on-chronic liver failure: real-world study

**DOI:** 10.3389/fmicb.2023.1185492

**Published:** 2023-05-25

**Authors:** Wenting Peng, Huimin Gu, Da Cheng, Keyu Chen, Cichun Wu, Chuan Jiang, Jinqing Liu, Shifang Peng, Lei Fu

**Affiliations:** Department of Infectious Diseases, Xiangya Hospital, Central South University, Changsha, Hunan, China

**Keywords:** hepatitis B virus, acute-on-chronic liver failure, tenofovir alafenamide, kidney function, efficacy

## Abstract

**Background and aims:**

Real-world data regarding hepatitis B virus-related acute-on-chronic liver failure (HBV-ACLF) patients receiving tenofovir alafenamide (TAF) as an antiviral drug are limited. Hence, we evaluated the efficacy and kidney safety of TAF among this population.

**Methods:**

A total of 272 HBV-related ACLF patients hospitalized at Xiangya Hospital of Central South University were enrolled in this retrospective research. All patients received antiviral therapy with TAF (*n* = 100) or ETV (*n* = 172) and comprehensive medical treatments.

**Results:**

Through 1:1 propensity score matching, 100 patients were finally included in each group. At week 48, the survival rates without transplantation of the TAF group and ETV group were 76.00 and 58.00%, separately (*P* = 0.007). After 4 weeks of treatment, the TAF treatment group exhibited a significantly decline in HBV DNA viral load (*P* = 0.029). The mean estimated glomerular filtration rate was apparently improved in the TAF group compared with the ETV group (TAF 5.98 ± 14.46 vs. ETV 1.18 ± 18.07 ml/min/1.73 m^2^) (*P* < 0.05). There were 6 patients in TAF group and 21 patients in ETV group with chronic kidney disease (CKD) stage progression ≥ 1. By contrast, the ETV treatment group has a greater risk of renal function progression in CKD 1 stage patients (*P* < 0.05).

**Conclusion:**

This real-world clinical study showed that TAF is more effective than ETV in reducing viral load and improving survival rate in HBV-ACLF patients and the risk of renal function decline is lower.

**Clinical trial registration:**

https://ClinicalTrials.gov, identifier NCT05453448.

## 1. Introduction

Hepatitis B virus (HBV) infection is associated with substantial economic and health burdens, with an estimated 257 million patients chronically infected and approximately 1 million of deaths every year in the world (Seto et al., 2018). Chronic hepatitis B virus infection can be manifested as asymptomatic infection, and can also lead to hepatitis, liver failure, cirrhosis and even hepatocellular carcinoma (Tada et al., 2015). Some patients may experience acute exacerbation of the HBV infection and progression to acute-on-chronic liver failure (ACLF), which has a high mortality rate in spite of substantial supporting and the use of a great quantity resources (Hernaez et al., 2017). Liver transplantation is a latent treatment election for most ACLF patients; hence, factors such as donor shortage and high cost restrict its clinical application. Hence, Early intervention and treatment are very important in patients with ACLF. Oral nucleos(t)ide analogue (NA) therapy can suppress HBV replication, which alleviate hepatic cell death and accordingly helps prevent liver damage or decompensation-related multi-organ complications (Arroyo et al., 2021). Major guidelines recommend the use of entecavir (ETV), tenofovir disoproxil fumarate (TDF), and tenofovir alafenamide fumarate (TAF) as first-line NAs for treating chronic hepatitis B (CHB), as these drugs are very important in anti HBV treatment (European Association for the Study of the Liver, 2017). However, TAF has been on the market for a short time in the world, ACLF guidelines do not currently recommend TAF.

It is reported that TDF can be hydrolyzed to tenofovir after absorption, which leads to high levels of circulating tenofovir, and long-term use will lead to kidney and bone toxicity, and this is particularly problematic in aging populations (Jung et al., 2022b). TAF is a tenofovir pro-drug, which is converted into the active form of tenofovir diphosphate (TFV-DP) *in vivo*, similar to TDF. Due to its unique characteristics, TAF can reduce the total body exposure of TFV by more than 90% at a dose of ≤25 mg (Chan et al., 2016). The Correlativity study showed that TAF has a low concentration of tenofovir in the circulation, which can reduce the drug load in the kidney and bone, which improves the safety of the kidney and bone (Murakami et al., 2015). Current guidelines therefore suggest selecting TAF or ETV over TDF in patients with renal changes such as estimated glomerular filtration rate (eGFR) < 60 ml/min or in patient undergoing hemodialysis (European Association for the Study of the Liver, 2017).

Most previous studies of TAF and TDF have focused on their efficacy and safety (Pilkington et al., 2020). To date, few studies have directly weighed up the effectiveness and renal safety between TAF and ETV. In a retrospective trial involving patients with treatment-naïve CHB, ETV had a higher risk of renal function damage than TAF (Jung et al., 2022a). As a result of TAF has been used in China for a short time and the clinical real-world research data is lacking. Particularly, there is a lack of data with regard to the impact of TAF on renal function in patients with HBV-ACLF, not only in China but worldwide. Thus, we conducted this clinical study to assess the safety and effectiveness of TAF in treating HBV-ACLF patients in China.

## 2. Patients and methods

### 2.1. Study design and patient selection

Acute-on-chronic liver failure, as defined by the Asian Pacific Association for the Study of the Liver (APASL), is acute hepatic insult manifesting as jaundice (a serum bilirubin level of ≥5 mg/dL) and coagulopathy [an international normalized ratio (INR) ≥ 1.5 or prothrombin activity < 40%] (Sarin et al., 2019). The exclusion criteria included:(1) Less than 18 years old; (2) history of end-stage renal disease or kidney transplantation; (3) combined with other liver diseases, such as alcoholic liver disease, non-alcoholic fatty liver disease, autoimmune liver disease, drug-induced liver injury, hepatolenticular degeneration or other viral infections (hepatitis A, C, and E virus or HIV infection);(4) pregnant or lactating; (5) concomitant with malignant tumor or other serious disease affecting survival time; (6) patients with missing data; and (7) follow-up period of <48 weeks. From May 2020 to June 2021, HBV-related ACLF patients hospitalized in the Xiangya Hospital Central South University were recruited for this retrospective study. A total of 272 patients were contained in the research and fell into the TAF group and the ETV group according to their choice of medication.

The protocol was approved by the Medical Ethics Committee of Xiangya Hospital Central South University (approval no. 202201022).

### 2.2. Treatment and follow-up

During the study period, all patients received anti-hepatitis B virus treatment with 25 mg of TAF (Gilead Sciences, Inc., Foster City, CA, USA) or 0.5 mg of ETV (Fujian Cosunter Pharmaceutical Co., Ltd, Fujian, China) once daily immediately after diagnosis of HBV-ACLF. Simultaneously, comprehensive medical treatments were provided, including rest, ordinary supportive treatment, energy and vitamin supplementation, supplementation of blood products, such as albumin and blood plasma, and treatment of latent complicating diseases. An artificial liver support system (plasma exchange or double plasma molecular absorption system) was applied based on the physician’s discreet decision. All participants returned to the hospital for follow-up visits every 4 to 48 weeks after the initiation of medication. The clinical outcomes (survival without liver transplantation, death or liver transplantation) of each participant and relevant follow-up indicators of the survivors were recorded.

### 2.3. Data collection

Clinical and laboratory data were collected during hospitalization and included clinical characteristics, routine blood test results [including hemoglobin (HB), platelets, white blood cells(WBC)], liver function tests [including alanine aminotransferase (ALT), aspartate aminotransferase (AST), and total bilirubin (TBIL)], renal function tests [including serum creatinine (Cr), blood urea nitrogen (BUN), and eGFR], coagulation function tests (including INR, prothrombin time, and prothrombin activity), electrolytes (serum sodium, potassium), HBV DNA quantification (<10 IU/ml), serological biomarkers and Child-Turcotte-Pugh (CTP) score. The model for end-stage liver disease (MELD) score was calculated using the following formula (Malinchoc et al., 2000): MELD = 3.8 × ln [total bilirubin (mg/dL)] + 11.2 × ln (INR) + 9.6 × ln [creatinine (mg/dL)] + 6.4. The Chinese group on the study of severe hepatitis B-ACLF IIs (COSSH-ACLFs IIs) score (Xiao et al., 2021): COSSH-ACLFs IIs score = 1.649 × ln (INR) + 0.457 × HE score (0 stage/1 score; 1–2 stage/2 score; 3–4 stage/3 score) + 0.425 × ln (neutrophil) + 0.396 × ln [total bilirubin (mg/dL)] + 0.576 × ln (serum urea) + 0.033 × age (Xiao et al., 2021).

### 2.4. Outcomes

The primary outcome measure was 48-week liver transplantation-free survival. The secondary outcome of this research was chronic kidney disease (CKD) progression. CKD staging is determined by using CKD-EPI equation and referring to the global renal disease improvement criteria (Stevens and Levin, 2013).

### 2.5. Statistical analyses

All analyses were performed using SPSS for Windows, version 25.0. Continuous variables were reported as mean ± standard deviation or median (interquartile range), whereas categorical variables were reported as percentages. The Student *t*-test and the rank sum test were used for comparisons of continuous variables, and the chi-squared test was used for comparisons of categorical variables. The propensity score matching (PSM) was applied to balance baseline differences (Austin, 2009). All statistical tests were 2-sided, and a *P*-value < 0.05 was considered statistically significant.

## 3. Results

### 3.1. Differences in clinical characteristics between the TAF group and ETV group

**FIGURE 1:**
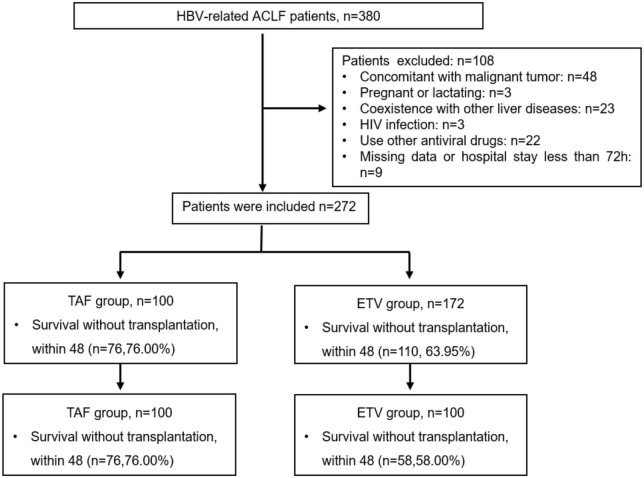
Flow chart of the patient inclusion process. HBV, hepatitis B virus; ACLF, acute-on-chronic liver failure; ETV, entecavir; TAF, tenofovir alafenamide.

From May 2020 to June 2021, our hospital found 380 patients with HBV-related ACLF, 108 of whom were excluded for various reasons (Figure 1). Finally, 272 patients were enrolled in the study, including 100 patients who underwent TAF therapy and 172 patients underwent ETV therapy. The median age was 47.43 ± 12.38 years, and 235 (85.40%) of the patients were male. A total of 86 patients died or underwent liver transplantation during the follow-up period. The clinical characteristics at baseline are shown in Table 1. Patients in TAF treatment group had higher HB, ALT, AST, HBV DNA levels, and HBsAg levels but lower age and BUN. There were no differences in the other indicators, including gender, total bilirubin Cr, INR, eGFR, MELD score, COSSH-ACLF II score, or CTP score. PSM was performed to balance the baseline factors, and finally 100 patients in TAF group and ETV group were included in the study. After the propensity score matching, the clinical data of the two groups are comparable. A total of 66 patients in this PSM cohort died or underwent liver transplantation during the follow-up period. The proportion of patients with hypertension (8.00 vs. 14.00%) and diabetes (15.00 vs. 17.00%) was statistically resemble between the TAF and ETV groups (all *P* > 0.05). They also receive relevant drug treatment according to their condition.

**TABLE 1:** Baseline characteristics of the study population.

	Unmatched			Matched		
**Variable**	**TAF group (*n* = 100)**	**ETV group (*n* = 172)**	***P*-value**	**TAF group (*n* = 100)**	**ETV group (*n* = 100)**	***P*-value**
Male, *n* (%)	84.00%	87.79%	0.379	84.00%	91.00%	0.134
Age (years)	45.22 ± 12.13	49.53 ± 12.3	0.015	45.22 ± 12.13	48.11 ± 12.85	0.055
WBCs (×10^9^/L)	6.08 ± 2.68	6.48 ± 4.09	0.529	6.08 ± 2.68	5.95 ± 2.3	0.775
N (×109/L)	4.17 ± 2.25	4.41 ± 2.5	0.656	4.17 ± 2.25	4.05 ± 2.05	0.626
HB (g/L)	140.31 ± 139.64	126.23 ± 78.9	0.043	140.31 ± 139.64	137.85 ± 100.27	0.666
PLTs (×10^9^/L)	116.13 ± 55.23	103.9 ± 56.09	0.054	116.13 ± 55.23	103.39 ± 50.62	0.100
Albumin (g/L)	30.64 ± 3.71	30.42 ± 4.13	0.534	30.64 ± 3.71	30.27 ± 3.66	0.300
Globulin (g/L)	28.91 ± 6.19	29.16 ± 7.09	0.645	28.91 ± 6.19	28.88 ± 5.91	0.699
TBIL (μmol/L)	338.55 ± 145.42	359.48 ± 179.7	0.625	338.55 ± 145.42	360.44 ± 169.78	0.711
DBIL (μmol/L)	189.28 ± 82.45	194.71 ± 92.14	0.713	189.28 ± 82.45	194.23 ± 84.1	0.839
ALT (U/L)	429.65 (200.85,876.78)	213.30 (89.35,517.85)	<0.001	429.65 (200.85,876.78)	424.45 (215.30,636.28)	0.641
AST (U/L)	303.05 (141.45,610.88)	180.00 (111.30,359.15)	0.001	303.05 (141.45,610.88)	270.25 (167.65,490.65)	0.780
BUN (mmol/L)	4.7 ± 3.41	6.04 ± 8.44	0.037	4.7 ± 3.41	4.72 ± 2.79	0.410
Creatinine (μmol/L)	91.93 ± 36.7	97.7 ± 72.21	0.443	91.93 ± 36.7	89.73 ± 22.66	0.570
eGFR (ml/min/1.73 m^2^)	89.13 ± 20.98	85.25 ± 24.08	0.141	89.13 ± 20.98	86.37 ± 19.64	0.158
Na (mmol/L)	137.98 ± 3.56	137.44 ± 3.71	0.135	137.98 ± 3.56	137.69 ± 2.96	0.280
Ca (mmol/L)	2.13 ± 0.12	2.14 ± 0.15	0.393	2.13 ± 0.12	2.13 ± 0.13	0.985
P (mmol/L)	0.77 ± 0.23	0.84 ± 0.29	0.252	0.77 ± 0.23	0.80 ± 0.26	0.837
PT (seconds)	22.38 ± 12.68	22.31 ± 9.03	0.553	22.38 ± 12.68	22.96 ± 9.93	0.228
INR	1.99 ± 1.17	1.98 ± 0.98	0.678	1.99 ± 1.17	2.04 ± 1.12	0.321
AFP (ng/ml)	146.35 (56.93,301.63)	114.00 (26.22,311.40)	0.109	146.35 (56.93,301.63)	138.70 (39.55,339.30)	0.988
HBV DNA log10 (IU/ml)	5.24 ± 1.66	4.68 ± 1.77	0.017	5.24 ± 1.66	5.02 ± 1.72	0.474
HBsAg log10 (IU/ml)	3.35 ± 1.11	3.1 ± 1.04	0.044	3.35 ± 1.11	3.20 ± 1.09	0.226
HBeAg-positive, *n* (%)	38.00%	30.23%	0.189	38.00%	34.00%	0.556
CTP score	10.40 ± 1.92	10.40 ± 2.00	0.994	10.4 ± 1.92	10.57 ± 1.98	0.490
MELD score	24.04 ± 5.86	24.49 ± 6.08	0.386	24.04 ± 5.86	24.61 ± 5.76	0.304
COSSH-ACLF IIs	5.56 ± 1.06	5.72 ± 1.05	0.124	5.56 ± 1.06	5.75 ± 1.06	0.127
Diabetes, *n* (%)	15.00%	13.37%	0.709	15.00%	17.00%	0.451
Hypertension, *n* (%)	8.00%	22.00%	0.224	8.00%	14.00%	0.175

Data are frequency (%), median M (P25, P75), or mean ± standard deviation. TAF, tenofovir alafenamide; ETV, entecavir; WBCs, white blood cells; PLTs, platelets; N, neutrophils; HB hemoglobin; ALT, alanine aminotransferase; AST, aspartate aminotransferase; TBIL, total bilirubin; DBIL, direct bilirubin; BUN, blood urea nitrogen; GFR, glomerular filtration rate; Na, natrium; Ca, calcium; P, phosphorus; PT, prothrombin time; INR, international normalized ratio; AFP, alpha fetoprotein; HBV, hepatitis B virus; HBsAg, hepatitis B surface antigen; HBeAg, hepatitis B e antigen; CTP, Child-Turcotte-Pugh; MELD, model for end-stage liver disease.

### 3.2. Survival rates without liver transplantation

**FIGURE 2:**
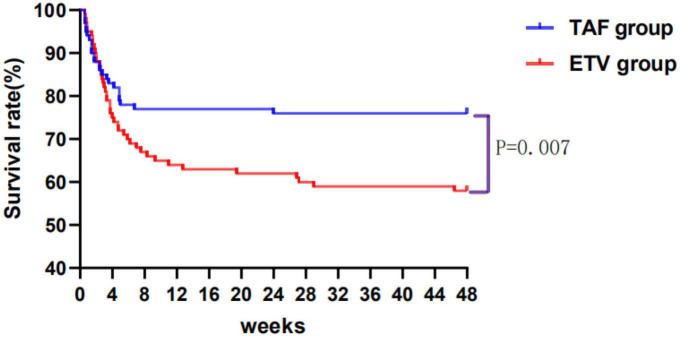
Cumulative survival of patients taking tenofovir alafenamide (TAF) or entecavir (ETV) over 48 weeks. TAF, tenofovir alafenamide; ETV, entecavir.

A total of 186 patients survived at 48 weeks of follow-up, with a survival rate of 68.38%, including 76 patients in TAF group and 132 patients in ETV group. The survival rates were similar between the TAF and ETV groups during follow-up. After the PSM, the survival rate of TAF was significantly higher than that of ETV group (all *P* < 0.05). As shown in Table 2, the survival rates of patients accepting TAF and ETV at week 4 were 85.00% and 75.00%, respectively. At week 12, their survival rates were 77.00% and 64.00%. At week 24, their scores were 76.00% and 60.00%, and at 48 weeks, the scores were 76.00% and 58.00%, respectively. Figure 2 shows the overall cumulative survival of patients in each group. It has been proven that TAF improves the survival rate of ACLF patients after 4 weeks of treatment, so we believe that TAF is more effective than ETV.

**TABLE 2:** Clinical outcomes of patients with HBV-related acute-on-chronic liver failure on tenofovir alafenamide or entecavir treatment.

Outcome, *n* (%)	TAF group (*n* = 100)	ETV group (*n* = 172)	*P*-value	TAF group (*n* = 100)	ETV group (*n* = 100)	*P*-value
**Survival rate (without transplantation)**						
Within 4 weeks	85 (85.00%)	132 (76.74%)	0.102	85 (85.00%)	75 (75.00%)	0.077
Within 12 weeks	77 (77.00%)	117 (68.02%)	0.114	77 (77.00%)	64 (64.00%)	0.044
Within 24 weeks	76 (76.00%)	114 (66.28%)	0.092	76 (76.00%)	60 (60.00%)	0.015
Within 48 weeks	76 (76.00%)	110 (63.95%)	0.039	76 (76.00%)	58 (58.00%)	0.007
**Liver-related complications**						
Spontaneous bacterial peritonitis	60 (60.00%)	113 (65.70%)	0.346	60 (60.00%)	69 (69.00%)	0.184
Hepatorenal syndrome	5 (5.00%)	18 (10.47%)	0.118	5 (5.00%)	11 (11.00%)	0.118
Hepatic encephalopathy	21 (21.00%)	27 (15.70%)	0.188	21 (21.00%)	20 (20.00%)	0.861
Ascites	64 (64.00%)	119 (69.17%)	0.379	64 (64.00%)	72 (72.00%)	0.225
Gastrointestinal hemorrhage	2 (2.00%)	6 (3.49%)	0.484	2 (2.00%)	3 (3.00%)	0.651

Data are frequency (%). TAF, tenofovir alafenamide; ETV, entecavir.

### 3.3. Biochemical and virological responses in the TAF and ETV groups

**FIGURE 3:**
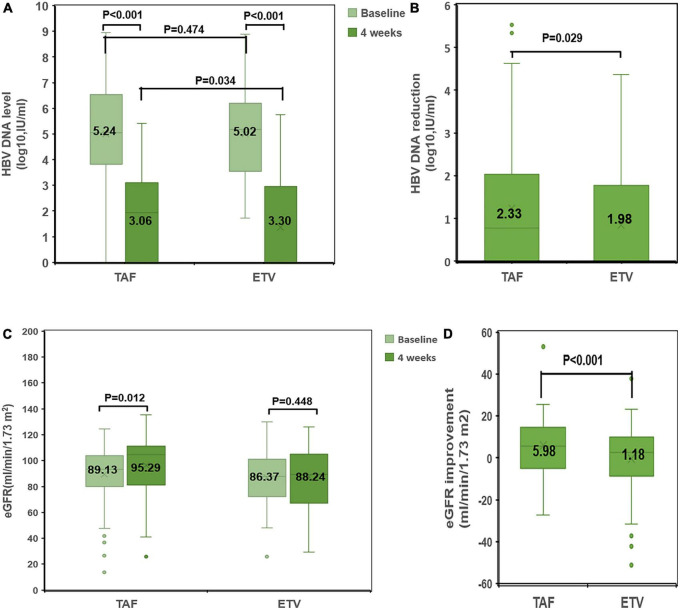
Comparison of changes in HBV DNA level and eGFR between the TAF and ETV groups. **(A)** HBV-DNA levels at baseline and at 4 weeks in the TAF and ETV groups. **(B)** HBV-DNA reduction from baseline to 4 weeks in the TAF and ETV groups. **(C)** eGFR levels at baseline and at 4 weeks in the TAF and ETV groups. **(D)** eGFR improvement from baseline to 4 weeks in the TAF and ETV groups. TAF, tenofovir alafenamide; ETV, entecavir; eGFR, estimated glomerular filtration rate; HBV, hepatitis B virus.

After treatment, the changes of ALT and albumin were obviously different in TAF group and ETV group. However, as shown in Table 3, there was no difference between the two groups. Similarly, no obvious differences were discovered in terms of INR, serum bilirubin, and MELD score between the two groups. Compared with the ETV group, the TAF group exhibited better virological response within 4 weeks. The carrying capacity of HBV-DNA decreased from 5.24 ± 1.66 to 3.06 ± 0.91 log_10_ (IU/ml) in the TAF group and from 5.02 ± 1.72 to 3.30 ± 0.89 log_10_ (IU/ml) in the ETV group (Figure 3). At 4 weeks, HBV DNA load was undetectable in 2 patients in TAF treatment group (2%; 2/100) and 1 patient in ETV treatment group (1%; 1/100) (*P* = 0.561). Nevertheless, the change in HBsAg in the two groups was similar.

**TABLE 3:** Index changes between the TAF and ETV groups after 4 weeks of treatment.

	TAF group (*n* = 100)	ETV group (*n* = 100)	*P*-value
**ALT**			
Before treatment	429.65 (200.85,876.78)	424.45 (215.30,636.28)	0.641
After 4 weeks	61.40 (43.80,88.00)	59.90 (41.60,86.50)	0.752
Reduction	368.25 (157.05,788.78)	315.50 (144.70,621.40)	0.484
P (baseline vs. 4 weeks)	**<0.001**	**<0.001**	
**TBIL**			
Before treatment	338.55 ± 145.42	360.44 ± 169.78	0.711
After 4 weeks	275.89 ± 191.94	293.29 ± 221.01	0.114
Reduction	66.62 ± 186.03	39.86 ± 189.47	0.512
P (baseline vs. 4 weeks)	**0.002**	**0.001**	
**Albumin (g/L)**			
Before treatment	30.64 ± 3.71	30.27 ± 3.66	0.300
After 4 weeks	34.42 ± 3.81	33.69 ± 4.08	0.338
Reduction	−2.06 ± 7.81	−4.22 ± 4.45	0.165
P (baseline vs. 4 weeks)	<0.001	<0.001	
**INR**			
Before treatment	1.99 ± 1.17	2.04 ± 1.12	0.321
After 4 weeks	1.88 ± 1.05	2.09 ± 2.24	0.564
Reduction	0.04 ± 1.06	-0.14 ± 2.07	0.940
P (baseline vs. 4 weeks)	0.276	0.318	
**MELD score**			
Before treatment	24.04 ± 5.86	24.61 ± 5.76	0.304
After 4 weeks	23.01 ± 8.61	23.66 ± 8.42	0.554
Reduction	1.27 ± 7.57	0.94 ± 6.68	0.648
P (baseline vs. 4 weeks)	0.252	0.300	

Median M (P25, P75). TAF, tenofovir alafenamide; ETV, entecavir; ALT, alanine aminotransferase; TBIL, total bilirubin; MELD, model for end-stage liver disease; INR, international normalized ratio. The bold values refer to the comparison between baseline data and data after 4 weeks of treatment.

### 3.4. Dynamic changes in renal function in the TAF and ETV groups

After treatment, Cr decreased significantly in TAF treatment group, and Cr decreased slightly in ETV treatment group. Within 4 weeks of treatment, there was a significant difference in the change in Cr between the two groups. Accordingly, eGFR in the TAF group increased significantly after treatment (eGFR: 89.13 ± 20.98 ml/min/1.73 m^2^ to 95.29 ± 20.12 ml/min/1.73 m^2^). In the ETV group, eGFR increased significantly after treatment (eGFR: 86.37 ± 19.64 ml/min/1.73 m^2^ to 88.24 ± 24.04 ml/min/1.73 m^2^) (Figure 3).

**FIGURE 4:**
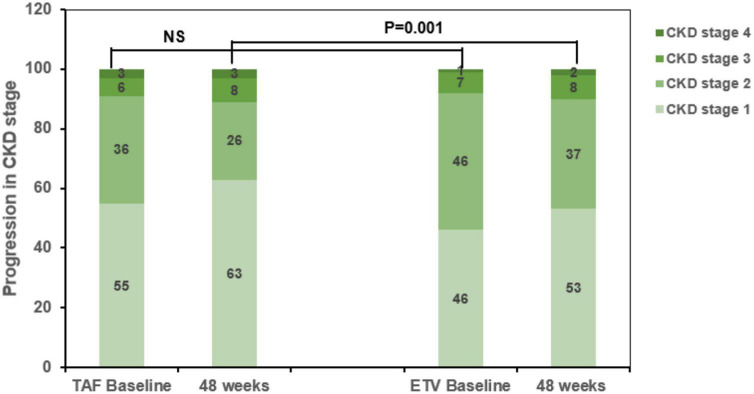
Progression of chronic kidney disease (CKD) stage among CKD progressors in the tenofovir alafenamide (TAF) and entecavir (ETV) groups. TAF, tenofovir alafenamide; ETV, entecavir; ALT, alanine aminotransferase; CKD, chronic kidney disease.

Kidney outcomes after PSM are shown in Figure 4. In the matched cohort, the baseline CKD stage of the two groups was divided into Stage 1, Stage 2, Stage 3, and Stage 4. In the TAF group, the stages included 55, 36, 6, and 3 patients, respectively. In the ETV group, the stages included 46, 46, 7, and 1 patient, respectively. Additionally, in TAF group and ETV group, there were 6 patients and 21 patients with renal function progression greater than stage one respectively.

Among the patients with CKD stage progression, 55 and 46 patients were in CKD stage 1 at baseline, respectively. Among them, 4 and 16 patients had renal function progression greater than stage 2 at the end point of the research in TAF treatment group and ETV treatment group. The incidence of ≥1 CKD stage in ETV group was significantly higher (*P* < 0.05, Figure 4).

### 3.5. Safety

During the 48 week follow-up period, no significant drug-related adverse reactions were perceived with the two oral antiviral drugs.

## 4. Discussion

Acute-on-chronic liver failure is a serious clinical syndrome with high short-term mortality. In Asia, hepatitis B virus infection is the main cause of ACLF. The pathogenesis of HBV-ACLF remains incompletely understood. Previous studies have shown that the severity of the disease is closely related to viral factors. Therefore, current guidelines recommend the immediate use of nucleos(t)ide analogues in patients with HBV infection (Sarin et al., 2019). We carried out a real world study.

Li et al. demonstrated that TAF and ETV treatment of HBV-ACLF are similar in the field of enhancing survival rate and improving virological response and liver function in China. In our study, the survival rate of TAF group was 85.00%, 2 patients received liver transplantation, and 13 patients died during the initial 4-week follow-up period, which was in line with the results of other nucleoside analogues reported in previous studies (Li et al., 2021). At week 48, the survival rate without liver transplantation in the TAF group was 76.00%, which was obviously higher than the ETV treatment group (58.00%). Compared with the ETV group, the ALT, TBIL, MELD scores and HBV DNA load in TAF group decreased with time in terms of liver function improvement and virological inhibition. Both the TAF group and ETV group exhibited significantly improved TBIL and ALT. In contrast to previous studies, the TAF group in our study exhibited a greater decrease in HBV-DNA than the ETV group within 4 weeks. The virological response of the TAF group was better than that of the ETV group within 4 weeks (Zhang et al., 2021). Early antiviral treatment can shorten and improve the symptomatic phase and facilitate clinical and biochemical improvement. The higher survival rate without liver transplantation in the TAF group compared with the ETV group could be because TAF inhibits virus replication and improves biochemical indicators more rapidly in the early stage. Relevant studies have indicated that virus replication drives the development and progression of HBV-ACLF. Therefore, inhibition of viral replication can reduce liver cell damage caused by an excessive immune response, thereby improving the survival rate of patients (Li et al., 2022).

The mortality of ACLF is related to the number of organ failure, and the prognosis of renal failure is the worst. According to APASL related studies, 23 to 64% of ACLF patients have renal dysfunction. In comparison with patients with decompensated liver disease, patients with acute-on-chronic liver failure have higher disease prevalence, more rapid progression of renal insufficiency and faster tubular injury progression, and higher mortality (Jha et al., 2013; Maiwall et al., 2015; Pati et al., 2016). Renal dysfunction is an stand-alone predictive factor of mortality in HBV-ACLF (Angeli et al., 2015). Therefore, clinicians should keep a close watch on the risk of renal damage when treating HBV-ACLF patients.

Patients being treated for HBV-ACLF are possible to also develop hepatorenal syndrome and have an increased danger of renal failure. Renal function in these patients is affected by age and other factors. Previous studies have shown that in patients with HBV ACLF, the eGFR value can be preserved up to 40 years old, which is defined as the age threshold for the onset of age-dependent renal decline. The average eGFR of young people (under 40 years old) was 107.3 ml/min/1.73 m^2^, and then decreased. The average decline rate between 40 and 100 years old was 0.92 ml/min/1.76 m^2^/year (Pottel et al., 2017). In addition to age, complications such as diabetes and hypertension can damage kidney function. When treating ACLF, the use of kidney-damaging drugs, inappropriate diuresis, and ascites drainage can cause renal dysfunction or even renal failure, which affects the survival of patients with HBV-ACLF (Chan et al., 2018; Lim et al., 2020). Relevant guidelines indicate that early assessment and prevention of declining renal function can improve patient prognosis (Bajaj et al., 2022).

Previous studies have shown that TAF and ETV exert similar effects on kidney function in patients. However, our study confirm that TAF can conspicuously decrease the risk of renal damage. There are some studies comparing the nephrotoxicity of TAF and TDF, but there is no study directly comparing the renal risk between TAF and ETV and studying the application in patients with renal insufficiency in patients with HBV ACLF. Current data indicate that patients with ACLF have more complications and higher CKD incidence rate than the general population. Chronic hepatitis B virus infection and nucleoside analogues treatment were markedly correlated with the progression of chronic renal diseases and osteoporosis/fracture (Wong et al., 2018).

As all NAs are excreted via the kidneys, clinicians should monitor for progression of renal dysfunction (Lo and Wong, 2014). The advantage of TAF in lowering the risk of renal damage makes it a kind choice for CHB patients with potential or related risk of renal damage. Early detection and treatment can usually prevent or delay adverse results caused by kidney damage. At present, EASL guidelines proposed using TAF when eGFR declines below 60 ml/min/1.73 m^2^ (European Association for the Study of the Liver, 2017). Therefore, we examined the progression of renal dysfunction in patients at different stages of renal failure.

Jung et al. (2022a) showed that TAF can improve bone and renal function effects related to renal failure, and patients with chronic hepatitis B viral infection who treated with ETV have a higher risk of renal function decline. In our study, significant differences were observed in the changes in Cr and eGFR between the TAF and ETV groups with 4 weeks of treatment. TAF had a more significant protective effect on the kidneys than ETV, which was in accordance with the results previously reported.

Our results showed that in patients with stage 1 CKD, fewer patients in the TAF group progressed to higher CKD stages compared with patients in the ETV group. The rates of progression to renal dysfunction of CKD 2, CKD 3, and CKD 4 were similar. These data show that the use of TAF can slow the progression of renal dysfunction in the early stage of kidney injury. At present, the mechanism underlying the superior renal safety profile of TAF compared with ETV remains unclear. A research by Lampertico et al. showed that disadvantageous variety in renal laboratory arguments during long-term TDF use were largely reversible when patients with eGFR > 50 ml/min/1.73 m^2^ switched to TAF (Lampertico et al., 2020). The present study confirmed that using antiviral drugs that have less effect on renal function can reverse renal injury in the early stages. The causes for the divergences between the results of different studies are not clear, but one feasible illustration is that patients with chronic renal insufficiency experience irreversible renal damage in the middle and late stages.

Our study had some limitations. First, from an ethical perspective, it is hard to conduct an ideal randomized controlled trial (RCT) for serious, life-threatening disease such as ACLF. Secondly, serum creatinine and eGFR were used as indicators to evaluate renal function in this study. However, based on the relevant research foundation of clinical pharmacology, the reliability of this study can be improved by adding indicators reflecting renal tubular function. Therefore, we will examine the effect on renal tubule function in a subsequent study. Finally, this study was conducted at a single center with a relatively limited sample; thus, a multi-center study with a larger sample size and longer follow-up of HBV-ACLF patients is needed to authenticate the results of our study.

## 5. Conclusion

In conclusion, our results indicate that TAF is better than ETV in improving survival and virological response in the treatment of HBV-ACLF in China and may have a lower the risk of renal damage. Multi center prospective research is expected to be carried out to verify our results.

## Data availability statement

The original contributions presented in this study are included in the article/supplementary material, further inquiries can be directed to the corresponding author.

## Ethics statement

This study was performed in accordance with the ethical standard of the Declaration of Helsinki and was approved by the Institutional Review Board. Consent waiver was granted as this was a clinical audit of treatment outcomes. The study protocol strictly followed the principles of the Helsinki Declaration, and the study has been registered at ClinicalTrials.gov (registration no. NCT05453448).

## Author contributions

LF designed the experiments and supervised the study. WP, KC, and HG collected the clinical data. WP, CW, and DC performed the experiments and wrote the manuscript. SP assisted in experiments. All authors critically reviewed the final manuscript.
